# Looking for a Silver Lining to the Dark Cloud: A Google Trends Analysis of Contraceptive Interest in the United States Post Roe vs. Wade Verdict

**DOI:** 10.7759/cureus.27012

**Published:** 2022-07-19

**Authors:** Priyankar K Datta, Sumit R Chowdhury, Ajisha Aravindan, Sayan Nath, Parijat Sen

**Affiliations:** 1 Anaesthesia, Critical Care and Pain Medicine, All India Institute of Medical Sciences, New Delhi, New Delhi, IND; 2 Neuroanaesthesiology and Critical Care, All India Institute of Medical Sciences, New Delhi, New Delhi, IND; 3 Critical Care Medicine, All India Institute of Medical Sciences, New Delhi, New Delhi, IND; 4 Pulmonary and Critical Care Medicine, University of Kentucky, Lexington, USA

**Keywords:** female reproductive health, sexual and reproductive health rights, contraception, public health, pregnancy, policy making, human rights, abortion

## Abstract

Background

In the wake of the recent Roe vs. Wade judgment, we performed a Google Trends analysis to identify the impact of this decision on the interests regarding contraceptive choices in the United States.

Methods

A Google Trends search between April 6 and July 5, 2022, with the United States as the area of interest, was performed using the five most popular contraception choices. In addition, a second trend search was performed using oral and injectable hormonal birth control measures.

Results

Trends showed a spike in interest regarding various contraceptive methods immediately following the verdict. The highest increase in interest was noted for “vasectomy,” followed by “tubal ligation.” With respect to oral and injectable birth control measures, “morning after pill” showed a marked spike in interest.

Conclusion

This verdict has triggered increased interest in contraceptive practices, which can be translated into better reproductive health with proper public health initiatives.

## Introduction

On June 24, 2022, the United States Supreme Court overturned its landmark 1973 verdict in the Roe vs. Wade legal suit that had established the constitutional right to medical abortion [1]. This decision, which sparked sharp reactions within the US and globally, could have deep social ramifications and potentially impact the reproductive health of millions of people, especially women [2,3]. Moreover, this ruling threatens to precipitate further the impending crisis of access to safe abortion care in the United States [4,5]. In the background of this watershed event, we performed a Google Trends analysis to identify the impact of this decision on search interests regarding contraceptive choices in the US.

## Materials and methods

A 90-day Google Trends search with the United States as the area of interest was performed from the period April 6 to July 5, 2022, using the five most popular choices for contraception in the US: permanent female sterilization, oral contraceptive pill, male condom, intrauterine device, and partner vasectomy [6]. A search was done using the following commonly used layman terms - “tubal ligation” for permanent female sterilization; “birth control pill” for oral contraceptive pill; “condom” for male condom; “IUD” for an intrauterine device; and “vasectomy” for partner vasectomy. A second trend search was performed using oral and injectable hormonal birth control measures - “birth control pill,” “morning after pill” for emergency contraceptive pill, and “birth control shot” for contraceptive injection. The exact point of the US Supreme Court verdict was identified on the timeline. As per the Google Trends algorithm, search interest was quantified on a normalized scale of 0-100, with the highest indicator assigned as 100 [7]. Spike in search interest was quantified as the ratio of maximum interest generated post-verdict compared to the interest on June 23, immediately prior to the verdict. State-wise breakdown of trends was done to determine which states generated the most interest regarding various contraceptive methods.

## Results

**Figure 1:**
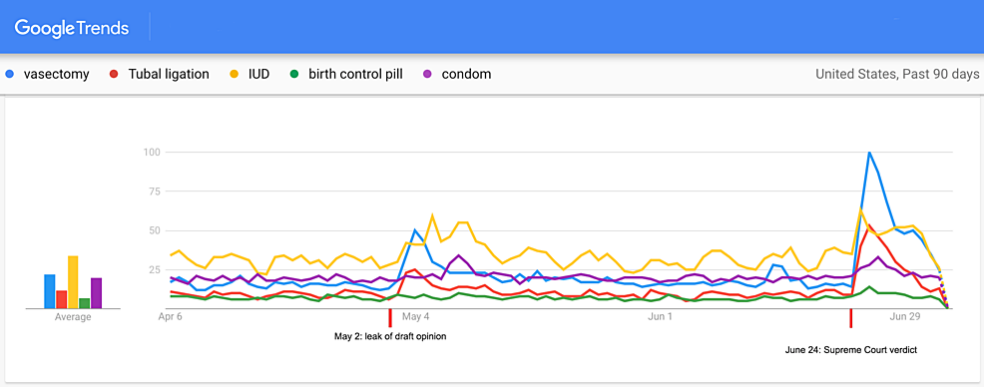
Google Trends search for popular contraceptive choices in the United States over the last 90 days (from April 6 to July 5, 2022).

**Figure 2:**
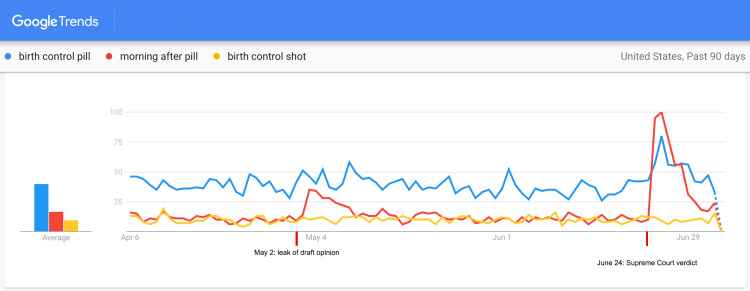
A 90-day Google Trends search (from April 6 to July 5, 2022) with respect to oral and injectable hormonal contraceptive techniques.

The past 90-day trend (from April 6, 2022 to July 5, 2022) showed a spike in interest regarding various contraceptive methods immediately following the verdict (Figure 1). Search traffic peaked between 24 and 72 hours after the verdict (June 24-June 26, 2022). Table 1 shows the magnitude of increase in search interest regarding various contraceptive methods following the verdict. Among the top five contraceptive choices in the United States, the highest increase in interest was noted for “vasectomy” (7.14 times), followed by “tubal ligation” (5.89 times), “IUD” (1.8 times), “condom” (1.75 times), and “birth control pill” (1.57 times). A smaller spike in internet search activity was noticed between May 2 and May 6, 2022. The second trends search performed using oral and injectable hormonal birth control measures (Figure 2) showed a marked spike in the interest in “morning after pill” (8 times) between 24 and 48 hours of the verdict. There was no noticeable increase in the interest in “birth control shot.” The geographical analysis of the trends showed variations in search interests among the states with respect to contraceptive choices (Table 2).

**Table 1:** The magnitude of the spike in interest for various contraceptive methods post-verdict compared to baseline (June 23, 2022) (quantified on a normalized scale of 0-100, with the highest indicator assigned as 100). IUD: Intrauterine device.

Contraceptive method	Baseline search interest on June 23	Peak search interest post-verdict	Spike ratio (peak/baseline)
Vasectomy	14	100	7.14
Tubal ligation	9	53	5.89
IUD	35	63	1.80
Birth control pill	8	14	1.75
Condom	21	33	1.57
Morning after pill	2	16	8.00

**Table 2:** Top five states with respect to interest in individual contraceptive choices. IUD: Intrauterine device.

Position	Vasectomy	Tubal Ligation	IUD	Birth control pill	Condom	Morning after pill
1	Oklahoma	Arkansas	Utah	Alaska	Delaware	Idaho
2	South Dakota	West Virginia	District of Columbia	Rhode Island	New York	District of Columbia
3	Idaho	Kentucky	Montana	New Hampshire	New Jersey	South Dakota
4	New Mexico	Mississippi	Colorado	Maine	Connecticut	Oklahoma
5	Hawaii	North Dakota	Minnesota	Connecticut	Mississippi	North Dakota

## Discussion

The Google Trends search indicates that there has been a recent spurt in interest regarding contraceptive methods following the Roe vs. Wade verdict. A similar, albeit smaller spike in internet search activity, was noticed following the “leak” of the draft opinion by Justice Alito in this matter on May 2, 2022 [8]. From the temporal association of these spikes with news regarding the verdict in question, we can infer that the increase in internet search interest is a direct consequence of the Supreme Court ruling regarding the ban on abortions.

Google Trends is an objective data source being increasingly used in health care research [9]. These trends have proven to be especially useful in gauging general awareness regarding public health issues [10,11]. The search trends included in the present article give valuable insight into the minds of US citizens on the background of the Roe vs. Wade verdict. They show which choices people find most convenient and are likely to adopt. It is apparent that most people impacted by this verdict appear to be now looking for long-term or permanent contraceptive choices (vasectomy and tubal ligation). Contrary to our expectations, we found only a modest increase in interest in the “birth control pill,” one of the safest and most effective means of birth control used extensively by women worldwide [12]. This apparent lack of interest in the “birth control pill” in relation to the other contraceptive methods may be attributed to a lack of awareness resulting in low confidence regarding efficacy, concerns regarding side effects, recurring costs, or simply inconvenience due to lack of over-the-counter availability in the US [13]. Similarly, the lack of interest in “birth control shots” may indicate a greater need for public education and awareness programs. The spike in interest in “morning after pill” is of concern. It points towards the prevalence of unprotected sexual practices and an over-reliance on the safety net of medical termination of unwanted pregnancy. In the US, emergency contraceptive pills are available over-the-counter, whereas regular contraceptive pills require a prescription [14]. This policy may need a serious rethink.

These trends may enable public health authorities to strategize outreach and dissemination of sexual health information regarding various contraceptive choices. Outreach programs should be specially focused on the most vulnerable social groups [15]. These trends may be utilized to tailor reproductive health initiatives to suit different states. They may also help gauge the expected burden of demand with respect to different contraceptive methods in the coming months.

## Conclusions

As authors, we respect an individual’s right to choose regarding his or her body. This verdict may be a dark cloud as it curtails this freedom. However, this has also triggered increased interest in contraceptive practices, which can be turned into a silver lining of better reproductive health with proper public health initiatives.
